# Activity of Wnt/PCP Regulation Pathway Classifies Patients of Low-Grade Glioma Into Molecularly Distinct Subgroups With Prognostic Difference

**DOI:** 10.3389/fonc.2021.726034

**Published:** 2021-09-01

**Authors:** Meng Zhang, Dan Wang, Lan Su, Jingjiao Ma, Sizhen Wang, Meng Cui, Shunming Hong, Bing Guan, Xiaodong Ma

**Affiliations:** ^1^Medical School of Chinese People’s Liberation Army, Beijing, China; ^2^Department of Neurosurgery, The First Medical Centre, Chinese People’s Liberation Army General Hospital, Beijing, China; ^3^Department of Neurosurgery, The Second Hospital of Southern District of Chinese People’s Liberation Army Navy, Sanya, China; ^4^Genetron Health (Beijing) Co. Ltd., Beijing, China; ^5^Department of Neurosurgery, The Third Medical Centre, Chinese People’s Liberation Army General Hospital, Beijing, China; ^6^Health Economics Department, Chinese People’s Liberation Army General Hospital, Beijing, China

**Keywords:** low-grade glioma, genomics, subtyping, prognosis, Wnt/PCP regulation

## Abstract

Wingless/Int-1 (Wnt) signaling is one of the most well-known oncogenic pathways. Numerous studies have uncovered an aberrant expression of Wnt in cancer and its association with multiple oncogenic processes, such as cell proliferation, epithelial–mesenchymal transition (EMT), and invasiveness. Most previous studies mainly focused on the canonical branch of Wnt signaling pathway, i.e., Wnt/β-catenin signaling. The Wnt/planar cell polarity (PCP) signaling pathway, as the most recently described branch of Wnt signaling, was much less investigated in oncology research. In this study, we thoroughly characterized the activity of the Wnt/PCP regulation pathway in low-grade glioma (LGG) patients. Subtyping based on the expression pattern of the Wnt/PCP regulation pathway revealed three (C1–C3) subgroups with significant survival differences. Each group displayed distinct genomic characteristics. For instance, C1 was enriched with capicua transcriptional repressor (CIC) truncating mutations and 1p19q codel. C2 was characterized with tumor protein p53 (TP53) and ATRX chromatin remodeler (ATRX) inactivating mutations but depletion of telomerase reverse transcriptase (TERT) promoter mutations. C3 showed elevated malignancy reflected from several oncogenic characteristics, such as tumor heterogeneity and cell stemness, and demonstrated the worst survival outcome. In addition, C3 showed elevated macrophage segregation *via* induction of cytokines that are able to enhance the permeability of the brain–blood barrier (BBB). Lastly, we developed a prognostic model based on the risk score system. Validation indicated that our model can independently predict the prognosis of LGG patients.

## Introduction

Low-grade glioma (LGG), as the name suggested, is a type of low-grade malignancy (as opposed to high-grade tumor, like glioblastoma) originated from glial cells. Histologically, it used to be roughly classified into three major types: astrocytoma, oligodendroglia, and oligoastrocytoma, a malignancy of mixed cell types (1, 2). Each subtype presents with different phenotypic characteristics. For instance, astrocytoma is prevalent in the younger population while oligodendroglia occurs primarily in adults and results in relatively better life expectancy (3, 4). Such traditional classification based on histology set the groundwork for guiding the treatment as well as prognosis for glioma since the 1920s (5).

With the advancement of biotechnology and its application in cancer research, we are able to expand our understanding of LGG through exploring its characteristics at the molecular level. A number of studies, such as The Cancer Genome Atlas (TCGA) project and Chinese Glioma Genome Atlas (CGGA), comprehensively analyze hundreds of LGG samples and reveal a full genomic spectrum of LGG (6). Numerous recurrent landmarks have been successively identified and investigated in-depth regarding their functional impacts. For instance, somatic mutations in isocitrate dehydrogenase (NADP(+)) (IDH) characterize the majority of adult LGG patients and define an LGG subtype with favorable prognosis (7). LGG with IDH mutation and 1p/19q codel often co-occurs in oligodendroglioma, which demonstrates better responses to chemoradiotherapy (8). Cyclin-dependent kinase inhibitor 2A/B (CDKN2A/B) homozygous deletion in IDH1/2 mutant astrocytoma associates with a poor prognosis, similar to WHO IV tumors (9). Other genomic events, such as mutations in CIC, far upstream element-binding protein 1 (FUBP1), and TERT promoter, is also found frequently mutated in patients with IDH mutations. The interplay of these hallmarks has been widely studied (10). The elucidation of the functional impact and phenotypical consequence of these molecular hallmarks has been facilitating the transition from histological classification to molecular classification of LGG in clinical settings (5).

The Wnt signaling pathway is known to play a pivotal role in embryonic development, including cell fate determination, cell proliferation, and cell migration (11). These processes are fundamental for tissue differentiation during embryonic development. Till today, three Wnt pathway branches are categorized: the canonical Wnt pathway, also known as Wnt/β-catenin pathway, and two noncanonical pathways: Wnt/Ca^2+^ and Wnt/PCP. The Wnt/β-catenin pathway is the most well-studied Wnt signaling branch. As its name suggested, the signaling transaction depends on the β-catenin accumulation in the cytoplasm. Its aberrant activity has been extensively linked to tumorigenesis by influencing cell fate, proliferation, and migration (12–15). The Wnt/Ca^2+^ pathway leads to the phospholipase C (PLC)-mediated release of Ca^2+^ into the cell, controls intracellular calcium levels, and activates protein kinase C (PKC), calcineurin, or calmodulin-dependent kinase II (16–18).

Wnt/PCP as the most recently recognized Wnt pathway branch has been much less characterized. The Wnt/PCP pathway was first described by Gubb and Garcia-Bellido in 1982 when studying a mutant of fruit fly presenting defects in orientation of hairs on the wings (19). Subsequent studies identify a number of genes that constitute what is now commonly called the Wnt/PCP pathway (20). The identified Wnt/PCP components are highly conservative across species implying its functional significance in maintaining normal cellular function (14). Similar to Wnt/β-catenin, Wnt/PCP is fundamental to embryotic development and Wnt/PCP dysfunction may lead to body axis malformation (21). One major difference is that Wnt/PCP is β-catenin independent. Instead, it activates G proteins, such as Rho and Rac, which are involved in different signaling processes including the c-Jun N-terminal kinase (JNK) pathway (22). The upregulation of Wnt/PCP activity has been observed in multiple cancer types, including breast cancer, melanoma, and colorectal cancer (23, 24). However, the functional significance of Wnt/PCP in LGG has not been thoroughly investigated (25). In this study, we mainly focused on the noncanonical Wnt/PCP pathway; therefore, the Wnt/PCP regulation pathway was also included. We leveraged genomic, transcriptomic, and epigenetic data of 510 LGG tissue samples. Through integrative multi-omics analysis, we unveiled three LGG subtypes of distinct molecular characteristics as well as life expectancy and explored the utility in clinical practice.

## Materials and Methods

### Data Collection

Integrative analyses were conducted on tumor tissue samples of LGG patients enrolled in the TCGA project. Raw expression data (gene expression and DNA methylation) as well as processed genomic alteration data including somatic mutations (SNVs) and somatic copy number alterations (SCNAs) of 510 LGG samples were retrieved from the Genomic Data Commons (GDC) data portal (https://portal.gdc.cancer.gov/). Principal component analysis (PCA) was performed to ensure that no apparent batch effect was involved. Due to the lack of normal control samples in the TCGA LGG dataset, we alternatively retrieved samples from the Genotype-Tissue Expression (GTEx) Project (https://www.gtexportal.org/home/). For all 13 anatomical regions within the brain in the GTEx project, we excluded samples within regions from where glioma rarely arises (cerebellum, cerebellar hemisphere, hypothalamus, spinal cord, hippocampus, and anterior cingulate cortex). A total number of 1,452 samples from the remaining seven regions were collected as normal control (frontal cortex, cortex, caudate, nucleus accumbens, putamen, substantia nigra, and amygdala).

### Bioinformatics and Statistical Analysis

Pathway enrichment analysis was performed using ssGSEA (v4.0). Gene-set annotation regarding pathways of interest was derived from the Molecular Signatures Database (https://www.gsea-msigdb.org/gsea/msigdb) and Pathway Common (https://www.pathwaycommons.org/). Recurrent SCNAs were identified using GISTIC2 (v2.0) (26). The correlation between continuous variables was measured using the Spearman correlation coefficient. The correlation between continuous variables and categorical variables was assessed using either the Mann–Whitney U test or the Kruskal–Wallis test depending on the number of categories. To test for statistical significance, the p-value was set to 0.05. Due to the large sample size in our study, we additionally estimated the effect size during comparison. Unsupervised clustering was performed using consensus clustering (ConsensusClusterPlus; v1.42.0) with the *k*-medoids algorithm, specifically Partitioning Around Medoids (PAM) and Spearman correlation as distance measurement. The optimum number of clusters was determined based on the delta area plot. Survival analysis was demonstrated using the Kaplan–Meier curve, and statistical significance was determined based on the log-rank test. The Cox proportional hazard model was used to adjust potential cofounders during multivariate survival analysis. In the dependency estimation of oncogenic events in LGG, we included point mutations, focal SCNAs, and arm-level SCNAs that are statistically enriched in specific LGG subtypes. Statistical significance was tested using Fisher’s exact test, and Bonferroni correction was applied for multiple-testing issues.

### Intratumoral Heterogeneity Estimation

Because intratumoral heterogeneity is known to reflect the overall genomic aberration of tumor tissue, a thorough evaluation regarding intratumoral heterogeneity was carried out from three perspectives: mutation burden, tumor ploidy, and clonality. Tumor mutation burden (TMB) was defined as the average amount of somatic mutations per Mb within the protein-coding region. Note that only somatic mutations were counted if it satisfied the following criteria: 1) allele frequency > 5%; 2) variant not included in common dbSNP of the dbSNP knowledgebase; and 3) population frequency of variant < 1% in 1000 Genome Project Phase 3. The aneuploidy score was estimated based on the method of Alison M Taylor to reflect the total amount of arm-level SCNA events (27). Briefly speaking, SCNA spanning over 80% of the chromosome arm was considered as arm-level SCNA event. Tumor clonality was estimated using PyClone (v0.13.0) (28).

### Quantitative Assessment of Molecular Characteristics of the LGG Subtype

To estimate cell stemness between LGG subtypes, we introduced mDNAsi from the study by Tathiane M. Malta et al. (29) to reflect the degree of stemness level of each LGG sample. Epithelial mesenchymal transition (EMT) is an important early event in metastasis formation by many cancers including gliomas, in which the Wnt/β-catenin signaling pathway has established roles (30, 31). EMT was estimated based on a 76-gene signature derived from a study by Lauren Averett Byers et al. (**Supplementary Table 1**) (32). For samples in each subtype, the expression matrix of these 76 genes was fetched into PCA. The eigenvector of the first PC was considered as EMT driven, and the corresponding eigenvalue was defined as the EMT score. The global methylation level of LGG samples was measured and compared between subtypes. The activity of two downstream signaling pathways (cytoskeleton and JNK signaling) was analyzed using enrichment analysis.

### Prognostic Model Construction Using the Risk Score System

To build a prognostic model predicting the likelihood of LGG patients being alive at 1, 3, and 5 years after diagnosis, all independent features were identified through stepwise multivariate Cox regression. The risk score was calculated by multiplying the feature value, and its corresponding coefficient is as follows:

$$Risk\;score=\sum_{i=1}^{n}\exp*\beta$$

where *n* is the total number of independent factors, *exp* is the feature value, and *β* is the regression coefficient of feature *i.* Intuitively, the risk score represents the weighted sum of all independent features based on Cox regression. The risk score was subsequently used in the regression model to assess the likelihood of corresponding patients being alive at given years after diagnosis. The model performance was evaluated using the receiver operating characteristic (ROC) curve on testing sets. Another dataset obtained from CGGA was used for independent validation.

## Results

### The Expression Spectrum of the Wnt/PCP Regulation Pathway Classifies LGG Subtypes With Differentiated Prognosis

**Figure 1:**
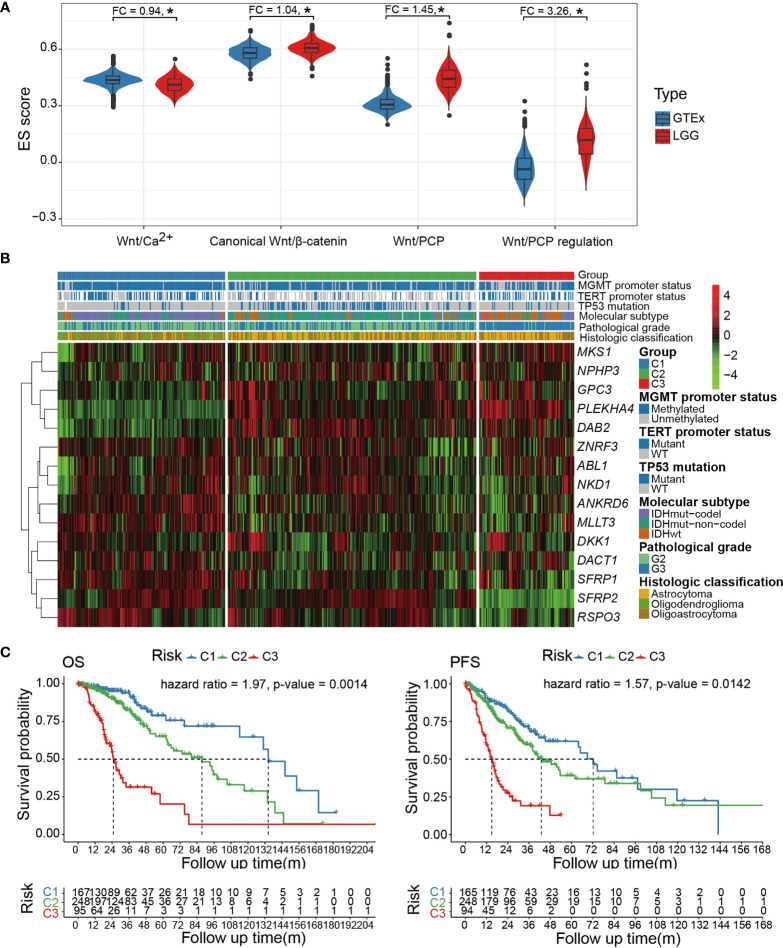
**(A)** Comparison of pathway enrichment for each of the four Wnt pathway branches (Wnt/β-catenin, Wnt/Ca^2+^, Wnt/PCP, and Wnt/PCP regulation). **(B)** The expression spectrum of the Wnt/PCP regulation pathway categorizes three LGG subtypes with distinct molecular characteristics as well as differentiated prognosis. **(C)** Cox proportional-hazard model indicated that the Wnt/PCP regulation pathway can independently predict survival of LGG patients. *P < 0.05.

To gain an overview of the Wnt signaling activity in LGG, we collected curated gene sets that represent each Wnt pathway branch (Wnt/β-catenin, Wnt/Ca^2+^, Wnt/PCP, and Wnt/PCP regulation). Since we mainly focused on the Wnt/PCP pathway, the Wnt/PCP regulation pathway was also included in our analyses (**Supplementary Table 2**). The expression enrichment of each pathway was estimated using ssGSEA and compared between LGG samples and normal brain tissue samples. Compared with the Wnt/β-catenin and Wnt/Ca^2+^ pathways, the Wnt/PCP and Wnt/PCP regulation pathway were much more differentially expressed with fold changes (FC) of 1.45 and 3.26, respectively (**Figure 1A**). We realized that the functional impact of genes in the Wnt/PCP regulation pathway is directional (either upregulates or downregulates the activity of the Wnt/PCP pathway). To further dissect the expression spectrum of the Wnt/PCP regulation pathway in LGG, we further performed unsupervised clustering using PAM. A total of 510 LGG samples were clustered into three groups marked as C1 (n = 167), C2 (n = 248), and C3 (n = 95) (**Figure 1B**). This surprisingly resembled with multi-omics (DNA methylation, DNA copy number, mRNA, and microRNA)-based subtyping in the study by the Cancer Genome Atlas Research Network (33) (**Supplementary Figure 1**), suggesting the central role of Wnt/PCP signaling in glioma progression. Furthermore, gene-wise clustering revealed three expression patterns (phylogenetic tree at the left side) by which C1 and C3 can be largely differentiated. Some genes, such as glypican 3 (GPC3), pleckstrin homology domain-containing A4 (PLEKHA4), DAB adaptor protein 2 (DAB2), ankyrin repeat domain 6 (ANKRD6), and secreted frizzled-related proteins (SFRPs), demonstrated an opposite expression between C1 and C3. A closer look into the gene function revealed that GPC3, PLEKHA4, DAB2, and ANKRD6 are positive regulators of the Wnt/PCP pathway. For instance, studies have shown that GPC3 can interact with Wnt and frizzled protein (FZD) and positively regulate downstream signaling (34, 35). On the other hand, SFRPs negatively regulate Wnt/PCP signaling by forming an inhibitory complex with frizzled protein (36). In brief, the expression pattern of core genes in the Wnt/PCP regulation pathway revealed in **Figure 1B** demonstrated the increasing activity of the Wnt/PCP pathway from C1 to C3.

In terms of oncogenic landmarks, Wnt/PCP regulation-based subtyping resulted in a similar classification where the C1 and C2 subtypes were dominated by IDH mutation & 1p/19q codel and IDH mutation & 1p/19q intact, respectively, whereas C3 was enriched with the IDH wild type. Besides, samples in C2 harbored significantly fewer mutations within the TERT promoter but significantly more mutations in TP53. In terms of pathological grade, C3 was enriched with samples of Grade 3 while most samples in C1 and C2 remained of Grade 2. It is reasonable to suspect that the survival difference determined by the log-rank test earlier may be biased by the imbalanced distribution of pathological grade as well as those genomic landmarks that are known to affect patient survival, such as IDH and 1p/19q status. We therefore included these potential confounders into Cox proportional-hazard model analysis and showed that the survival difference remains significant with hazard ratios of 1.97 and 1.57 [overall survival (OS) and progression-free survival (PFS)], demonstrating the independence of the Wnt/PCP regulation pathway as a prognostic factor (**Figure 1C**). This was successfully reproduced in another 408 LGG samples from CGGA to demonstrate the robustness of the Wnt/PCP regulation pathway in LGG prognosis (**Supplementary Figure 2**).

### An Insight Into Genomic Alterations of Each Wnt/PCP Regulation Subtype

**Figure 2:**
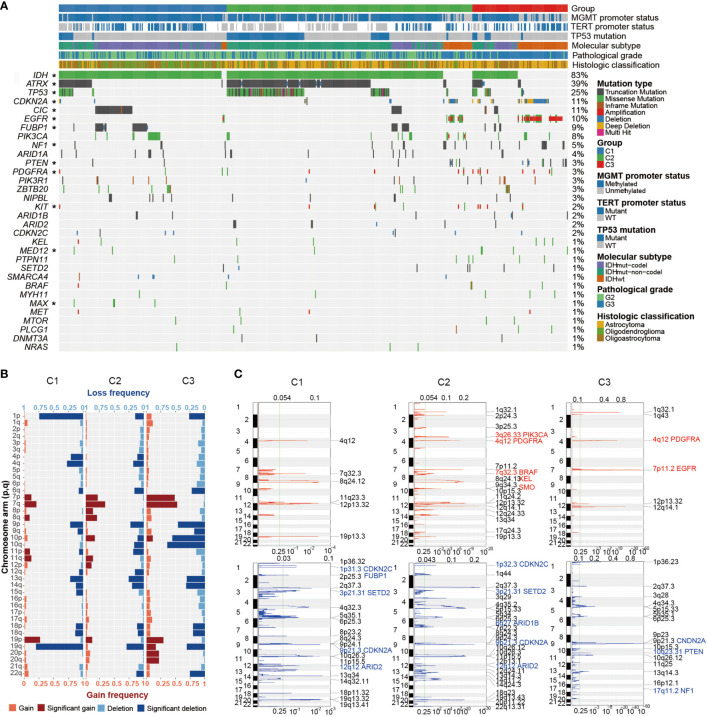
The genomic profile of LGG. **(A)** Mutational landscape of LGG. LGG samples were subtyped based on the expression spectrum of the Wnt/PCP regulation pathway. Mutation types are color labeled. Significant enrichments of specific mutations are asterisked at the left side. **(B)** Landscape of arm-level and focal SCNAs. Recurrent arm-level SCNAs were depicted in each group; the horizontal axis indicates the proportion of samples harboring corresponding arm-level SCNA event, and the vertical axis represents the arm of each chromosome. Both copy number gain (red) and loss (blue) were further classified into shallow and deep copy number alteration. **(C)** Recurrent focal SCNAs were depicted in each group. Dashed line marks the q-value threshold of 0.25. Known driver SCNAs are marked with the gene name. *P < 0.05.

We thought to investigate the genomic characteristics of each Wnt/PCP group. **Figure 2A** displays the mutational overview labeled by the Wnt/PCP group. IDH1 and IDH2 were collapsed together due to the similarity of their functional impact on glioma development. Note that we only displayed driver genes in LGG defined in the study by Francisco Martínez-Jiménez et al. (37). Each Wnt/PCP regulation subtype showed a distinct landscape of mutations (**Figure 2A**). In C1, CIC-inactivating mutations, as a landmark of oligodendroglioma (10), were dominant and showed mutual exclusive patterns to ATRX mutation, suggesting an alternative evolutionary branch. In C2, ATRX was significantly mutated as the known landmark of astrocytic glioma. Together with frequent TP53-inactivating mutation and IDH mutation, this combinatorial maker has been used to distinguish astrocytic glioma from oligodendroglioma (38, 39). In C3, IDH mutations were significantly depleted whereas epidermal growth factor receptor (EGFR) amplification and CDKN2A deletion were significantly enriched.

In terms of SCNAs, there were several known alterations as well as unexpected difference in terms of arm-level SCNAs between Wnt/PCP regulation subtypes (**Figure 2B**). As expected, the 1p/19q codel correlated with samples of oligodendroglioma origin in C1 (40, 41). The SCNA spectra of the other chromosomes were similar between C1 and C2. However, C3 displayed higher aneuploidy characterized with elevated gains of chromosomes 7, 19, and 20 and losses of chromosome 9, 10, 13q, 14q, and 19q.

To gain a higher resolution of copy number changes in each group, we also constructed recurrent focal SCNAs (**Figure 2C**). Driver SCNAs were marked according to the study by Francisco Martínez-Jiménez et al. (37). For instance, EGFR and phosphatase and tensin homolog (PTEN) were recurrently changed in their copy number within chromosomes 7 and 10, respectively. Homozygous copy number loss of CDKN2A was shared across groups. It has been proposed to promote tumor progression by enabling cellular senescence bypass (42). The oncogene smoothened (SMO) specific to C2 has been proposed to transduce Hedgehog signaling (SSH) from tumor suppressor patched 1 (PTCH1) to glioma-associated oncogene GLI family zinc finger (GLI) transcription factors and promote glioma tumorigenesis (43). By integrating point mutations, focal SCNAs, and arm-level SCNAs, we displayed pairwise dependency of major oncogenic events in LGG (**Supplementary Figure 3**).

### C3 Subtype Demonstrates High Intratumoral Heterogeneity

**Figure 3:**
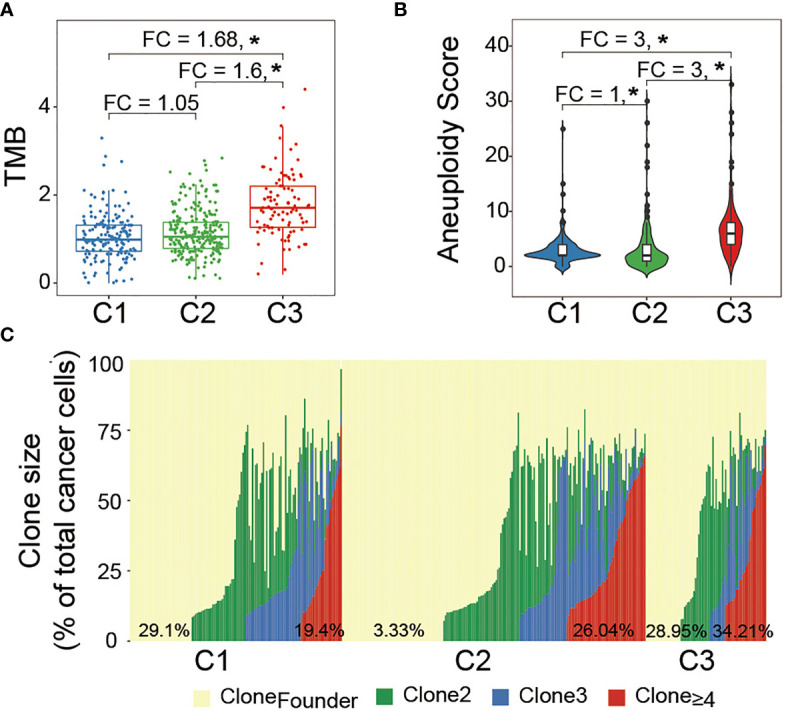
Intratumoral heterogeneity comparison between LGG subtypes: **(A)** median TMB was compared between groups. Statistical significance was determined by the Mann–Whitney U test. **(B)** Median aneuploidy score was compared between groups. **(C)** Major clone (Clone 1) laid at the background and subsequent subclone was superimposed upon the previous clone. The horizontal axis represents each LGG sample while the vertical axis represents the cellular fraction of each clone marked by a different color for the corresponding LGG sample. The percentage at the bottom indicates the fraction of LGG samples belonging to each clone. *P < 0.05.

An intratumoral heterogeneity comparison was made between Wnt/PCP regulation subtypes. As shown in **Figure 3A**, the median TMBs were 1.02, 1.07, and 1.71 for C1, C2, and C3, respectively. C3 harbored significantly more somatic mutations than C1 and C2, although brain tumor in general harbors relatively few somatic mutations compared with other cancer types. **Figure 3B** indicates higher tumor aneuploidy in C3, consistent with the SCNA landscape displayed in **Figure 2B**. Several studies have proposed that the aberrant activity of Wnt/PCP signaling may disrupt the microtubule assembly and subsequently lead to chromosomal instability (CIN) (44–46); however, their interactive route largely remains mysterious. Moreover, tumor clonality estimated by PyClone showed that samples in C3 had apparently higher fraction samples (34.21% vs. 26.04% and 19.4%) harboring subclones greater than three (**Figure 3C**). Together, we demonstrated that, compared with C1 and C2, samples in C3 were characterized with higher tumor heterogeneity.

### C3 Subtype Demonstrated Aggressive Oncogenic Behavior

**Figure 4:**
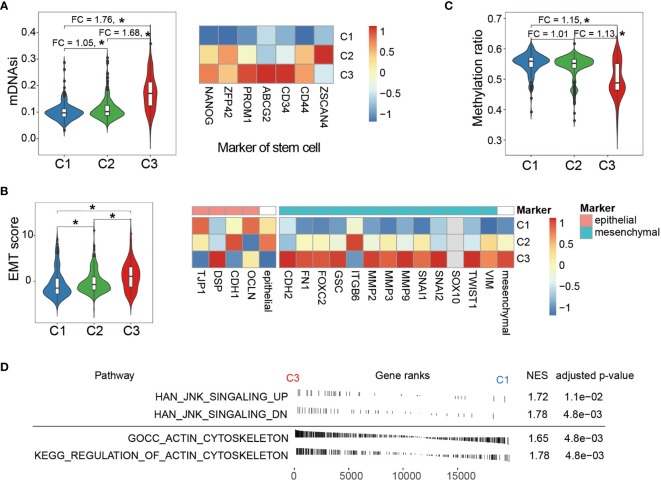
Comparison of phenotypic characteristics between LGG subtypes. **(A)** C3 shows significantly higher mDNAsi (left panel), an index reflecting cell stemness. The fold change of mDNAsi between groups was labeled at the top. Comparison of expression of cell stemness marker genes between the LGG groups (right panel). **(B)** EMT score comparison between the LGG groups (left panel). Comparison of expression of EMT marker genes shows severe tendency toward mesenchymal transition in the C3 group (right panel). Expressions of marker genes were taken by average and are shown at the right side of the epithelial and mesenchymal panel. **(C)** Comparison of global methylation levels indicated that C3 displayed extended de-differentiation. Fold changes between the LGG groups were labeled at the top. **(D)** Enrichment analysis showed that LGG samples in C3 exhibited elevated activity in both cytoskeleton and JNK signaling. NES indicates normalized enrichment score. *P < 0.05.

Cell stemness is one of the hallmarks during the development of many cancer types. As **Figure 4A** left panel displays, C3 showed significantly higher mDNAsi, and a comparison using the marker genes of cell stemness (right panel) demonstrated the same trend from C1 to C3. Cell stemness and EMT process are closely linked since cancer stem cells (CSCs) often express EMT characteristics (47–49). As expected, C3 also showed an extensive tendency toward mesenchymal transition (**Figure 4B**). Moreover, global methylation level, as one of the major characteristics reflecting cell stemness, displayed significant demethylation in C3 (**Figure 4C**). These observations altogether consistently suggested that samples in C3 gained many stem-like properties through extensive EMT which enhanced cell invasiveness and further contributed to tumor progression (50–53).

In addition, we explored the downstream impact of aberrant activity of the Wnt/PCP regulation pathway across LGG subtypes. It is known that Wnt/PCP signaling recruits Dishevelled (Dsh), which further regulates cytoskeleton (13, 21). Besides, Dsh can also form a complex with Ras-related C3 botulinum toxin substrate 1 (rac1) and subsequently activate JNK signaling (54, 55). These two downstream signaling pathways were analyzed using enrichment analysis between C1 and C3. As shown in **Figure 4D**, both signalings were significantly enriched in the C3 group. According to the modern cytoskeletal model, the drastic changes in cytoskeleton can subsequently promote cell migration (56, 57). In terms of JNK signaling, although its direct impact on tumorigenesis has not been fully delineated, JNK signaling activation has been observed in many cancer types and is likely to exert its oncogenic impact through multiple processes including cell differentiation, cell proliferation, and survival (58, 59).

### Immune Landscape of LGG

**Figure 5:**
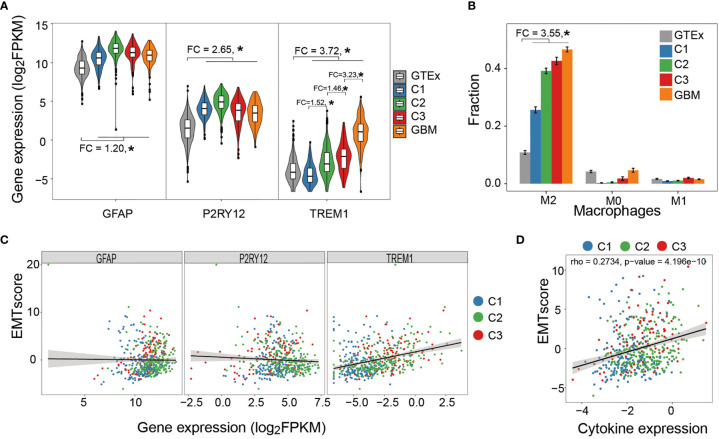
Correlation between immune cell infiltration and tumor stemness. **(A)** Astrocyte and microglia segregate toward tumor lesion in a tumor grade-independent manner while macrophage infiltration positively correlated with tumor grade. **(B)** Relative abundance of macrophage subtypes in healthy brain tissue (GTEx), LGG subtypes, and GBM samples. Macrophage M2 was the dominant subtype and demonstrated increasing abundance from healthy tissues to tumor samples. **(C)** Sample-wise correlation between immune cell infiltration and tumor stemness showed that only macrophage infiltration negatively correlated with tumor stemness. **(D)** Sample-wise correlation between tumor stemness and average expression of cytokines that affect BBB permeability. *P < 0.05.

Glial fibrillary acidic protein (GFAP), a marker gene of astrocyte, is one of the major immune effector cells in the brain. Compared with normal tissue, the higher expression of GFAP in LGG and glioblastoma (GBM) suggested a tendency of astrocyte segregation toward tumor lesion (**Figure 5A**). We further measured the expressions of purinergic receptor P2Y12 (P2RY12) and triggering receptor expressed on myeloid cells 1 (TREM1), previously reported markers that exclusively express in microglia and macrophage, respectively (60). The expression of P2RY12 showed a similar pattern with GFAP whereas TERM1 showed an elevated expression from normal tissue to each LGG subtype and to GBM (**Figure 5A**). Further cellular decomposition using CIBERSORT (61) revealed that macrophage M2 was dominant and, as expected, showed the same trend across different types of tissues (**Figure 5B**). In addition, sample-wise analysis showed that the expression of TREM1, rather than GFAP or P2RY12, significantly correlated with EMT score, indicating that macrophage M2 infiltration moderately correlated with enhanced tumor stemness (**Figure 5C**). Further analysis showed a moderate correlation between EMT score and the average expression of a number of cytokines (interleukin-6 (IL-6), interferon beta (IFN-β), transforming growth factor beta (TGF-β), granulocyte-macrophage colony-stimulating factor (GM-CSF), B-cell-activating factor (BAFF), interleukin-1 beta (IL-1-β), and tumor necrosis factor (TNF) known to be able to increase BBB permeability (**Figure 5D**).

### Constructing a Prognostic Predictor

**Figure 6:**
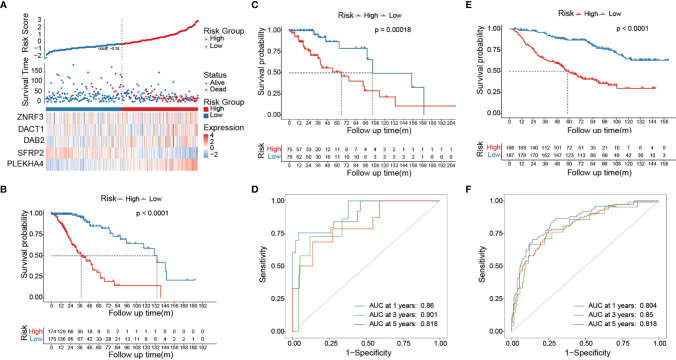
Risk score system predicts survival of LGG patients. **(A)** The ranked risk score of 357 LGG samples is shown at the top. Dot plot in the middle represents the survival time of the corresponding sample. Heat map shows the Log2-transformed expression of each prioritized gene. The vertical dashed line in the middle classifies LGG samples into low risk (blue) and high risk (red). **(B)** The Kaplan–Meier curve at the bottom showed survival difference between low-risk and high-risk groups. The Cox model was constructed on 357 LGG samples in the training set. The Cox model showed significant survival difference between the low-risk and high-risk groups. The p-value was calculated by the log-rank test. The validation was carried out on 153 and 408 LGG samples from the TCGA testing set **(C, D)** and CGGA dataset **(E, F)**. The AUROC curve demonstrates the performance of our model in predicting the survival of LGG patients.

**Figure 7:**
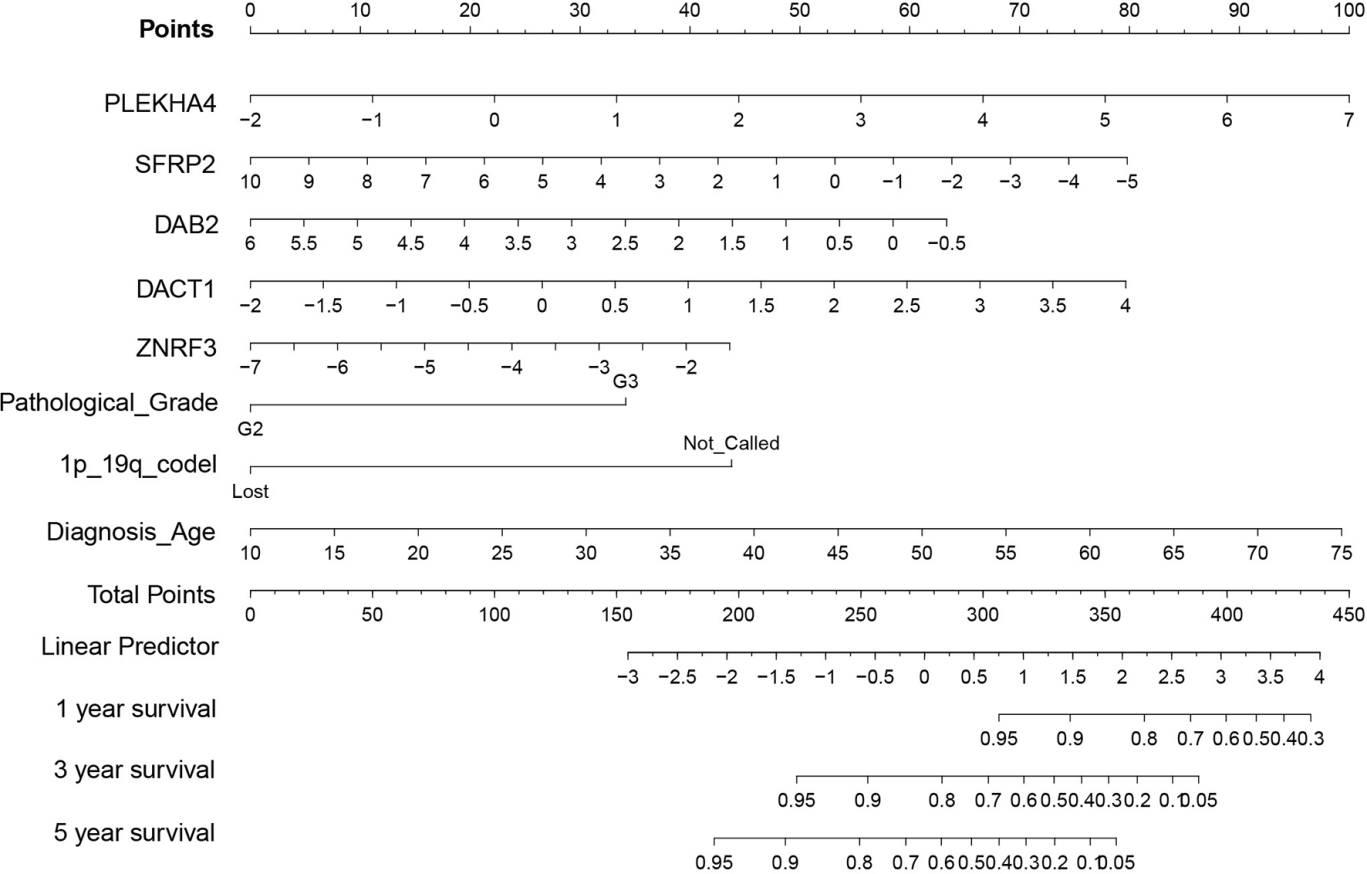
Nomogram of prognostic mode. Given prioritized features and the Cox model, a nomogram consisting of eight variables (PLEKHA4, SFRP2, DAB2, DACT1, ZNRF3, pathological grade, 1p/19q codel, and diagnosis age) was constructed for convenient clinical use.

To explore the prognostic value of Wnt/PCP regulation-based subtyping in clinical practice, we constructed a prognostic model predicting the probability of patients being alive at 1, 3, and 5 years after diagnosis. A total number of eight variables (PLEKHA4, SFRP2, DAB2, dishevelled binding antagonist of beta catenin 1 (DACT1), zinc and ring finger 3 (ZNRF3), pathological grade, 1p/19q codel, and diagnosis age) were prioritized by the stepwise multivariate Cox model and were used to construct a risk score system (see *Methods*). All 510 LGG samples were divided into a training set (n = 357) and a testing set (n = 153). In the training set (**Figure 6A**), sample risk was ranked alone with Log2-transformed expression of the corresponding gene. LGG patients were divided into low- and high-risk groups based on the median risk score of -0.28, and the Cox model showed a significant survival difference with a p-value less than 0.001. The median survival time for the low- and high-risk groups was 38 and 134 months, respectively (**Figure 6B**). This prognostic model was validated in the testing set (**Figure 6C**). The predictive model achieved areas under the curve (AUC) of 0.86, 0.9, and 0.82 for 1, 3, and 5 years, respectively, in the testing set (n = 153) (**Figure 6D**). To evaluate the robustness of our model, another independent validation was carried out on 408 LGG samples retrieved from CGGA and demonstrated an above 0.8 accuracy for all the 1-, 3-, and 5-year predictions (**Figures 6E, F**). Lastly, we constructed a nomogram for convenient clinical use (**Figure 7**).

## Discussion

### A Promising Role of the Wnt/PCP Regulation Pathway in LGG

The Wnt signaling pathway has been linked to carcinogenesis since the 1980s. However, most of the studies were conducted on the canonical Wnt/β-catenin pathway branch. Over the years, Wnt/β-catenin has received most of attention in investigating its physiological function as well as pathological involvement (62). Briefly, Wnt/β-catenin exerts its physiological function by acting as a stabilizer of β-catenin, an armadillo-repeat cytoplasmic adaptor protein, which, as a coactivator of the transcription factor, promotes the expression of Wnt-responsive genes (63). Normally, the cytoplasmic concentration of β-catenin is controlled by a number of regulators, including adenomatous polyposis coli (APC). In pathological conditions, the aberrant activity of Wnt/β-catenin prevents β-catenin from degradation (63). The β-catenin accumulation may subsequently promote the expression of Wnt-responsive genes and eventually lead to excessive stem cell renewal, a typical cancer hallmark. This oncogenic route is particularly well documented in colorectal cancer. Based on current oncology research, the same oncogenic process is now assumed to take place in a variety of cancer types. In glioma tissues, β-catenin expression is significantly higher than in normal tissues, and it positively correlates with the grade of gliomas (64–66). A high level of beta-catenin is also associated with poor prognosis in glioblastoma patients (67).

Our study mainly focused on the noncanonical Wnt/PCP pathway and Wnt/PCP regulation pathway. Through extensive multi-omics analyses, we found that, compared with other Wnt pathway branches, the noncanonical Wnt/PCP regulation pathway was more significantly upregulated, suggesting its greater role in the development of LGG. Based on the expression pattern of the Wnt/PCP regulation pathway, we demonstrated that the different extents of Wnt/PCP activity classify LGG into three subtypes with distinct molecular characteristics as well as life expectancy. In the prognostic analysis, we included potential confounders into Cox proportional hazards to eliminate the possible confounding effect of landmarks since the survival difference determined by the log-rank test may be biased by the imbalanced distribution of pathological grade as well as those genomic landmarks that are known to affect patients’ survival, such as IDH and 1p/19q status. We proved that our Wnt/PCP regulation-based subtyping can stratify patients with differentiated life expectancy. Additional independent datasets further confirmed the robustness of our model. The significance of this study is that, by measuring the activity of a single oncogenic pathway, we were able to predict LGG patients’ survival with relatively acceptable accuracy. Through a feature selection technique, the optimized model only consisted of the expression of five genes (PLEKHA4, SFRP2, DAB2, DACT1, ZNRF3) in addition to pathological grade, 1p/19q codel, and diagnosis age, offering an opportunity for developing a cost-effective approach for prognostics of LGG patients in clinical practice.

### Each Wnt/PCP Regulation Subtype Shows Special Genetic Associations or Mutually Exclusive Features

C3 subtype analysis showed an interesting pattern between EGFR, CDKN2A, and IDH in the perspective of the Wnt/PCP regulation pathway. In glioma, a number of oncogenic landmarks have been identified previously, such as somatic alternations on IDH, O-6-methylguanine-DNA methyltransferase (MGMT) promoter, TERT promoter, and chromosome 1p/19q co-del (7, 68). We found that Wnt/PCP regulation subtypes disproportionally distributed somatic mutations in the TERT promoter, ATRX, IDH, CIC, 1p/19q codel, and TP53. Moreover, based on the allele frequency and mutual dependency between these molecular landmarks, we were able to identify subtype-specific molecular events and explore the association of these events to phenotypic outcome. On the one hand, hotspot mutation in IDH, as the most prevalent genomic event in LGG, has been recognized as oncogenic *via* widespread disruption of histone modification and DNA demethylation (69, 70). Glioma formed from this evolutionary path has better prognosis and improved response to chemotherapy (71, 72). IDH was mutated in 83% of LGG samples with an average allele frequency of 0.35 (**Figure 2A**) in this study. The high population frequency as well as allele frequency is consistent with its initiative oncogenic nature in LGG. On the other hand, the overexpression of EGFR has been viewed as a highly malignant hallmark that significantly enhances cell proliferation *via* several signaling transductions, such as mitogen-activated protein kinase (MAPK), AKT serine/threonine kinase (Akt), and JNK (73). Several studies have revealed the convergence of EGFR and Wnt signaling through a reciprocal regulation at transcription as well as posttranscriptional level and suggested their collaborative contribution to the poor prognosis of glioma (74). Admittedly, most of the studies were conducted on the cross talk between EGFR and canonical Wnt signaling pathway. However, in this study, EGFR amplification and CDKN2A deletion seem to specifically occur in IDH wild-type samples in C3 (**Figure 2**). The mutual exclusion pattern between EGFR, CDKN2A, and IDH suggested that EGFR amplification and CDKN2A deletion, although less prevalent, act as initiative events alternative to IDH mutation in a small subset of LGG. Hence, this mutual exclusion implied an alternative evolutionary trajectory during the early stage of LGG development on the ground of the Wnt/PCP regulation pathway. Actually, there have been studies indicating the cross talk between EGFR and Wnt/PCP pathway and collaborative contribution to glioma progression (74, 75). However, the clear route of their cross talk largely remains mysterious.

In addition, we observed another two dependency modes. One was CIC-inactivating mutation combined with the 1p/19q codel, and the other was ATRX inactivating mutation combined with TP53 inactivating mutation. These two modes were largely mutually exclusive and enriched in C1 and C2, respectively (**Figure 2**). Several studies have reported improved survival for patients harboring CIC-inactivating mutation (76). However, its functional involvement in tumor progression remains to be elucidated.

### The C3 Subtype Is More Oncogenic Than the C1 and C2 Subtypes

Our results support that patients of the C3 subtype are prone to have a poor outcome. We found that the C3 subtype presented with higher aneuploidy, elevated tumor heterogeneity, and more aggressive cellular behavior. First, the elevated copy number gain of chromosome 7 and the loss of chromosome 10 (7+/10-) have been speculated as the initiative events prevalent in IDH wild-type astrocytoma (77). The primary suspicion of their oncogenic involvement has been focused on the oncogene and tumor suppressor within the corresponding chromosome (78). Second, the increasing tumor heterogeneity facilitates tumor fitness, promotes tumor progression, and leads to poor survival outcome (79–81). Third, the C3 subtype displays high tumor plasticity. In the context of cancer cell plasticity, stemness and EMT processes both influence the functional state of cancer cells (82). Overexpression of various EMT transcription factors were reported to contribute to the stemness in cancer cells (83, 84). In this study, the C3 subtype was distinct in terms of these two processes. Fourth, it is known that epigenetics involves the formation and function of CSCs and affects their plasticity *via* changing DNA methylation and chromatin (85). Since epigenetic mutations are considered to withdraw the constraints imposed to keep cellular plasticity under control, the low level of methylation in this study hints that C3 subtype cells might carry more epigenetic mutations when compared with C1 and C2. Fifth, the Wnt/PCP-related downstream pathways, cytoskeleton and JNK signaling pathway, were enriched in the C3 subtype, implying enhanced cellular capability to proliferate and migrate. All these oncogenic characteristics together confer a worst prognosis of LGG patients in C3 subtype.

### An Alien Immune Landscape of LGG

Wnt/PCP signaling has been involved in cell stemness known as one of the cellular characteristics (86, 87). Previous pan-cancer studies described a relatively weak correlation between cancer cell stemness and immune cell infiltration (88). Thus, we measured GFAP expression to compare the immune activity between Wnt/PCP regulation subtypes and tried to figure out if the above correlation could be observed in brain tumor. Microglia is another major component in brain immune environment, but it is difficult to distinguish microglia from macrophage which may pass BBB and migrate to tumor lesion. To evaluate them separately, we also measured expressions of P2RY12 and TREM1. The result suggested that, while both specialized immune cell types (astrocyte and microglia) in the brain segregate around tumor lesion in a tumor grade-independent manner, macrophage infiltration positively correlates with tumor grade.

However, the immune landscape is discrepant to some extent. At the pan-cancer scale, the study by Kreso et al. showed a negative correlation between immune signature and cell stemness as well as intratumoral heterogeneity. They proposed that, for most cancer types, cell stemness leads to high intratumoral heterogeneity through immunosuppression and these intrinsic properties collaboratively enhance tumor fitness, eventually leading to poor prognosis (89). Interestingly, LGG is one of the few exceptions that showed a positive correlation between immune signature and cell stemness in their study. Similarly, our results showed a positive correlation between cell stemness and the expression of TERM1, a marker gene of macrophage, although GFAP and P2RY12 were insignificant. Our subsequent analysis on the expression of cytokines also suggested that high cell stemness and/or tumor intratumoral heterogeneity is immunogenic and can recruit peripheral macrophage. Therefore, this interesting and alien immune landscape of LGG is noticeable. We consider that this distinction from most cancer types may root in the specialty of an immuno-oncological interaction in the neuroimmune system. One possible explanation for such positive correlation might be that, during the interaction with the neuroimmune system, tumor cells of high stemness induce certain molecules that cause elevated BBB permeability and permit macrophage infiltration. However, our analysis only revealed the correlation between tumor intrinsic property and macrophage infiltration. Further experiments are needed to investigate their causative relationship. Although this positive correlation hints a potential therapeutic approach in the future, such response does not yet confer better survival of LGG patients in this study.

## Conclusions

In this study, the activity of the Wnt/PCP regulation pathway effectively classifies LGG into three distinct subtypes with significant prognostic difference. The Wnt/PCP regulation subtype of LGG varies in gene alterations, especially regarding aneuploidy, tumor heterogeneity, oncogenic behavior, and immune landscape. Combined with little clinical information, Wnt/PCP regulation subtyping indicates an available and robust approach of survival prediction for LGG patients in clinical practice.

## Data Availability Statement

Genomic data and clinical information of 510 LGG patients are available at the GDC data portal (https://portal.gdc.cancer.gov/). Raw expression data of healthy LGG samples are available at Genotype-Tissue Expression (GTEx) Project (https://www.gtexportal.org/home/). Raw expression data are available at CGGA (http://www.cgga.org.cn/download.jsp) and GSE61374 (https://www.ncbi.nlm.nih.gov/geo/query/acc.cgi?acc=GSE61374) and were used from model validation.

## Ethics Statement

Molecular and clinical data used in the study were collected from the public depository and ready for academic research use.

## Author Contributions

MZ, DW, and LS conceived the study, analyzed the data, and drafted the manuscript. JM and SW participated in the study design and data collection and provided bioinformatics as well as statistical consultation during the study. MC and SH participated in the data analysis and model construction. BG and XM as academic supervisor oversaw the research. All authors contributed to the article and approved the submitted version.

## Conflict of Interest

Authors DW and LS work as full-time employees at Genetron Health (Beijing) Co. Ltd. Authors JM and SW were employed by Genetron Health (Beijing) Co. Ltd.

The remaining authors declare that the research was conducted in the absence of any commercial or financial relationships that could be construed as a potential conflict of interest. 

## Publisher’s Note

All claims expressed in this article are solely those of the authors and do not necessarily represent those of their affiliated organizations, or those of the publisher, the editors and the reviewers. Any product that may be evaluated in this article, or claim that may be made by its manufacturer, is not guaranteed or endorsed by the publisher.

## Supplementary Material

Supplementary Figure 1 Circos plot of LGG sample subtype according to our method and method used in Cancer Genome Atlas Research Network et al.’s study. LGG samples were subtyped using our method as well as method used in Cancer Genome Atlas Research Network et al.’s study. Linkage showed in circos plot indicated that most samples were assigned into same group by both methods.

Supplementary Figure 2 Multivariate Cox regression of LGG patients in CGGA (n=408). Multivariate Cox regression showed survival difference between LGG subtype defined by Wnt/PCP regulation activity. The dashed line indicates median survival time of corresponding group.

Supplementary Figure 3 Pairwise dependency of major events in LGG Each one of the major genomic event in LGG was tested for co-occurrence (green) and mutual exclusion (purple) with others. Group was marked for subtype specific dependency. Statistical significant was marked based on pre-defined p-value cutoff.

Supplementary Table 1 List of 76-gene signature that represents Epithelial–mesenchymal transition (EMT) The gene list was derived from Lauren Averett Byers et al.’s study and used to estimate tendency of EMT of each LGG sample.

Supplementary Table 2 Signature genes involved in each of four Wnt pathway branches (Wnt/β-catenin, Wnt/Ca2+, Wnt/PCP and Wnt/PCP regulation). Genes in each list were used to estimate the expression enrichment of corresponding pathway.

## Abbreviations

Akt, AKT serine/threonine kinase; ANKRD6, ankyrin repeat domain 6; APC, adenomatous polyposis coli; ATRX, ATRX chromatin remodeler; AUC, area under curve; BAFF, B-cell-activating factor; BBB, brain–blood barrier; CDKN2A/B, cyclin-dependent kinase inhibitor 2A/B; CGGA, Chinese Glioma Genome Atlas; CIC, capicua transcriptional repressor; CIN, chromosomal instability; CSCs, cancer stem cells; DAB2, DAB adaptor protein 2; DACT1, dishevelled binding antagonist of beta catenin 1; Dsh, Dishevelled; EGFR, epidermal growth factor receptor; EMT, epithelial–mesenchymal transition; FC, fold change; FUBP1, far upstream element binding protein 1; GBM, glioblastoma; GDC, Genomic Data Commons; GFAP, glial fibrillary acidic protein; GLI, GLI family zinc finger; GM-CSF, granulocyte-macrophage colony-stimulating factor; GPC3, glypican 3; GSC, glioma stem cell; GTEx, genotype-tissue expression; IDH, isocitrate dehydrogenase (NADP(+)); IFNb, interferon beta; IL-1b, interleukin-1 beta; IL-6, interleukin-6; JNK, c-Jun N-terminal kinase; LGG, low-grade glioma; MAPK, mitogen-activated protein kinase; MGMT, O-6-methylguanine-DNA methyltransferase; OS, overall survival; P2RY12, purinergic receptor P2Y12; PAM, Partitioning Around Medoids; PCA, principal component analysis; PCP, planar cell polarity; PFS, progression-free survival; PKC, protein kinase C; PLAGL2, PLAG1-like zinc finger 2; PLC, phospholipase C; PLEKHA4, pleckstrin homology domain-containing A4; PTCH1, patched 1; PTEN, phosphatase and tensin homolog; rac1, Ras-related C3 botulinum toxin substrate 1; ROC, receiver-operator characteristic; SCNAs, somatic copy number alterations; SFRPs, secreted frizzled-related proteins; SMO, smoothened; SNVs, somatic mutations; TCGA, The Cancer Genome Atlas; TERT, telomerase reverse transcriptase; TGFb, transforming growth factor beta; TMB, tumor mutation burden; TNF, tumor necrosis factor; TP53, tumor protein p53; TREM1, triggering receptor expressed on myeloid cells 1; Wnt, Wingless/Int-1; ZNRF3, zinc and ring finger 3.
