# Supplementary figures and images for: Activity of Wnt/PCP Regulation Pathway Classifies Patients of Low-Grade Glioma Into Molecularly Distinct Subgroups With Prognostic Difference
Source: Front Oncol. 2021 Sep 1;11:726034. doi: 10.3389/fonc.2021.726034 (PMC8440981; doi:10.3389/fonc.2021.726034)

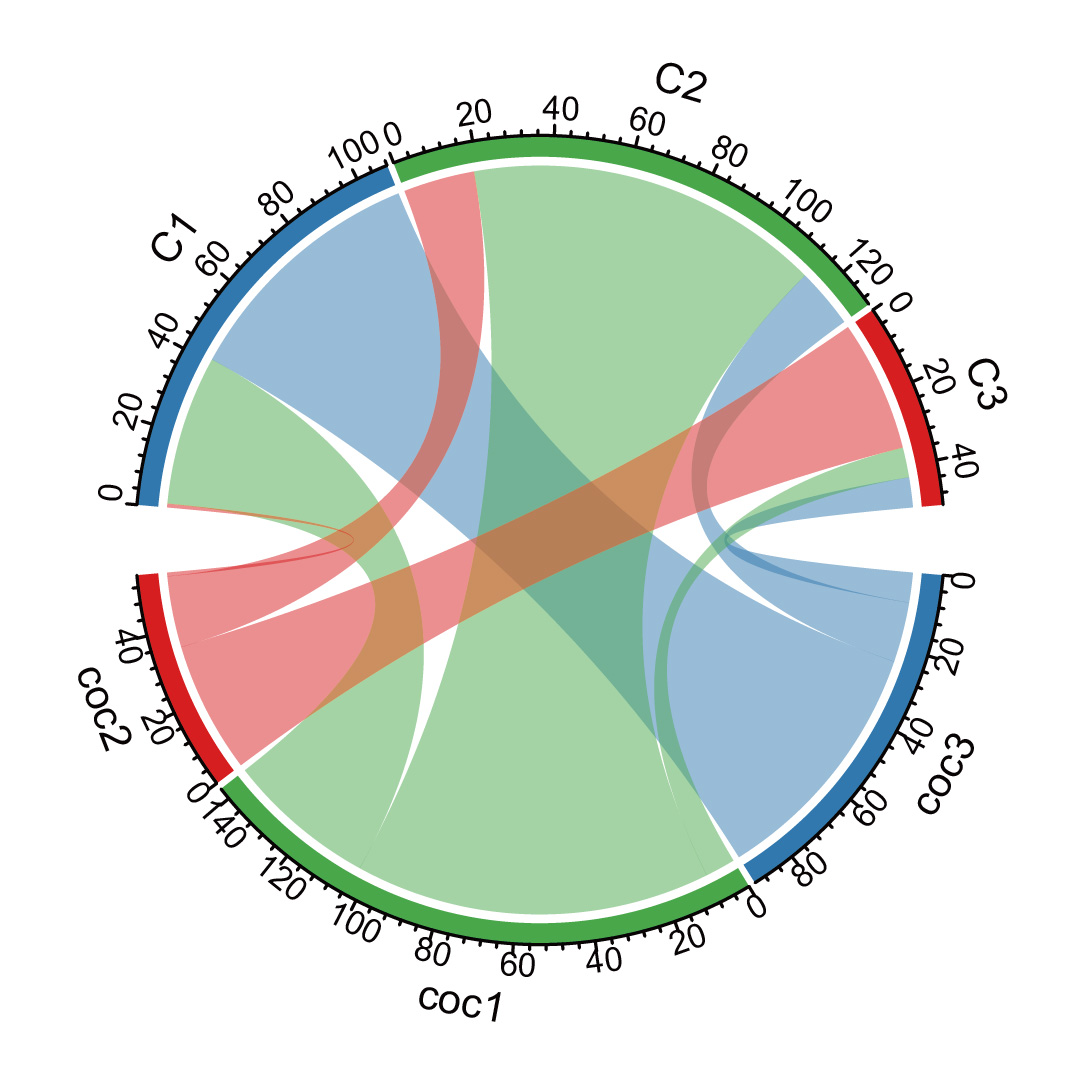

Supplement: Supplementary Figure 1 — Circos plot of LGG sample subtype according to our method and method used in Cancer Genome Atlas Research Network et al.’s study. LGG samples were subtyped using our method as well as method used in Cancer Genome Atlas Research Network et al.’s study. Linkage showed in circos plot indicated that most samples were assigned into same group by both methods. [file Image_1.jpeg]

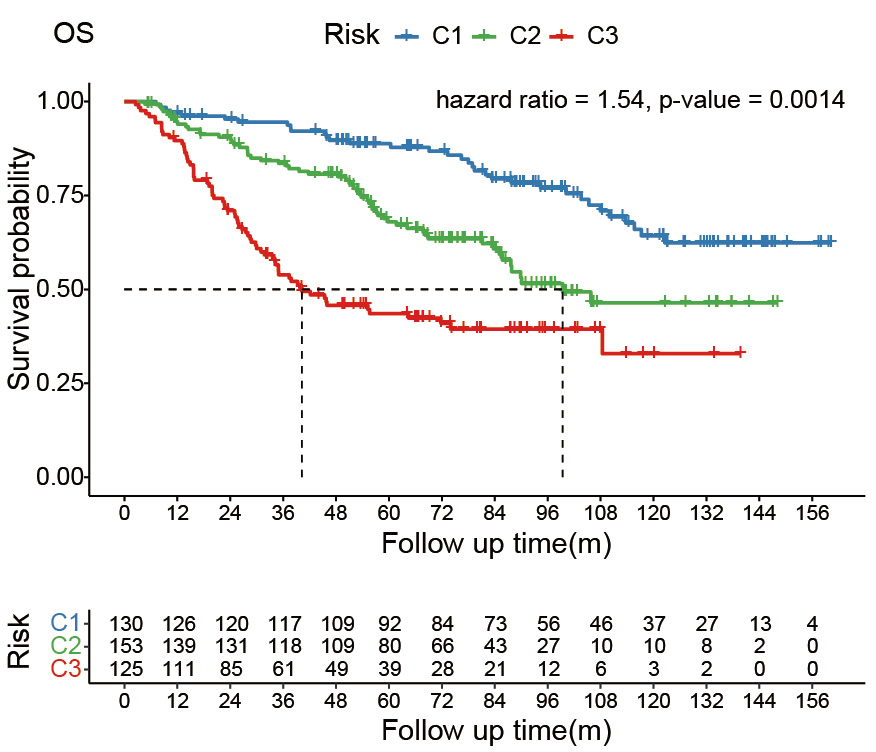

Supplement: Supplementary Figure 2 — Multivariate Cox regression of LGG patients in CGGA (n=408). Multivariate Cox regression showed survival difference between LGG subtype defined by Wnt/PCP regulation activity. The dashed line indicates median survival time of corresponding group. [file Image_2.jpeg]

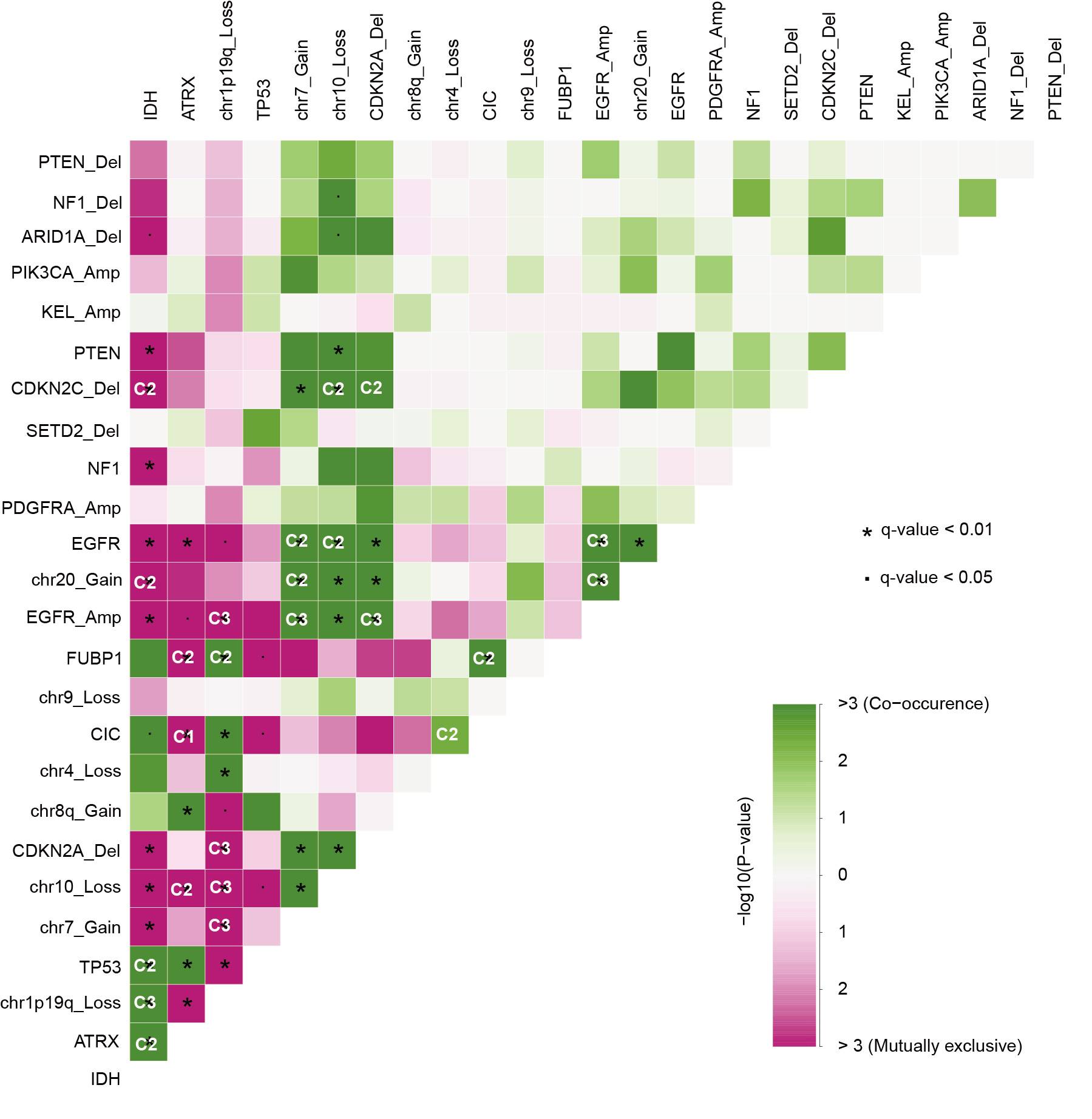

Supplement: Supplementary Figure 3 — Pairwise dependency of major events in LGG Each one of the major genomic event in LGG was tested for co-occurrence (green) and mutual exclusion (purple) with others. Group was marked for subtype specific dependency. Statistical significant was marked based on pre-defined p-value cutoff. [file Image_3.jpeg]
