# Supplementary material for: Activity of Wnt/PCP Regulation Pathway Classifies Patients of Low-Grade Glioma Into Molecularly Distinct Subgroups With Prognostic Difference
Source: Front Oncol. 2021 Sep 1;11:726034. doi: 10.3389/fonc.2021.726034 (PMC8440981; doi:10.3389/fonc.2021.726034)
Supplement: Supplementary Table 1 — List of 76-gene signature that represents Epithelial–mesenchymal transition (EMT) The gene list was derived from Lauren Averett Byers et al.’s study and used to estimate tendency of EMT of each LGG sample. [file DataSheet_1.pdf]

Supplementary Table 1: List of 76-gene signature that represents Epithelial–mesenchymal transition (EMT)

PRSS22	TGFBI	ANTXR2	LIX1L
VIM	AP1M2	CDS1	
SH3YL1	CRB3	TC2N	
CDH1	PPARG	AXL	
EPN3	CARD6	TMC4	
PRSS8	RAB25	SPINT2	
CDH3	PATJ	EVPL	
ERBB3	GRHL1	SERINC2	
GRHL2	GALNT5	KRT19	
MMP2	SHROOM3	SSH3	
FXYD3	TNFRSF21	XXYLT1	
DSP	EPHA1	KDF1	
NRP1	MAL2	STAP2	
ELMO3	ZEB1	TMEM125	
ESRP1	ST14	CLDN7	
KLC3	MPZL2	TMEM30B	
TJP3	MPP7	TSKU	
SCNN1A	HNMT	C1orf116	
ENPP5	TMEM45B	TACSTD2	
EPB41L5	ANKRD22	MUC1	
ITGB6	ADGRF1	PRR5	
GALNT3	MAPK13	CLDN4	
FN1	RBPMS	S100A14	
BSPRY	KRTCAP3	ADGRG1	
EPCAM	F11R	EPPK1	
