# Supplementary material for: Activity of Wnt/PCP Regulation Pathway Classifies Patients of Low-Grade Glioma Into Molecularly Distinct Subgroups With Prognostic Difference
Source: Front Oncol. 2021 Sep 1;11:726034. doi: 10.3389/fonc.2021.726034 (PMC8440981; doi:10.3389/fonc.2021.726034)
Supplement: Supplementary Table 2 — Signature genes involved in each of four Wnt pathway branches (Wnt/β-catenin, Wnt/Ca2+, Wnt/PCP and Wnt/PCP regulation). Genes in each list were used to estimate the expression enrichment of corresponding pathway. [file DataSheet_2.pdf]

Supplementary Table 2: Signature genes involved in each of four Wnt pathway branches (Wnt/β-catenin, Wnt/Ca2+, Wnt/PCP and Wnt/PCP regulation)

**Wnt/Ca2+**			**Canonical Wnt/β-catenin**	**Wnt/PCP**	**Wnt/PCP regulation**
Ca2+_pathway_Reactome			Canonical_Wnt_signal_NCI	PCP_CE_pathway_Reactome	GO_REGULATION_OF_WNT_SIGNALING_PATHWAY_PLANAR_CELL_POLARITY_PATHWAY
> Ca2+ pathway [http://identifiers.org/reactome/R-HSA-4086398]; datasource: reactome; organism: 9606; idtype: hgnc symbol			> Canonical Wnt signaling pathway [http://pathwaycommons.org/pc12/Pathway_cb80f0022b93ffbc95f93ebca756170b]; datasource: pid; organism: 9606; idtype: hgnc symbol	> PCP/CE pathway [http://identifiers.org/reactome/R-HSA-4086400]; datasource: reactome; organism: 9606; idtype: hgnc symbol	> Any process that modulates the frequency, rate or extent of Wnt signaling pathway, planar cell polarity pathway. [GOC:BHF]
AGO1	GNG11	PDE6B	APC	DAAM1	ABL1
AGO2	GNG12	PDE6G	AXIN1	DVL1	ANKRD6
AGO3	GNG13	PLCB1	CAV1	DVL2	DAB2
AGO4	GNG2	PLCB2	CSNK1G1	DVL3	DACT1
AXIN2	GNG3	PLCB3	CTNNB1	FZD1	DKK1
CALM1	GNG4	PPP3CA	CUL3	FZD3	GPC3
CAMK2A	GNG5	PPP3CB	DVL1	FZD6	MKS1
CTNNB1	GNG7	PPP3R1	DVL2	FZD7	MLLT3
FZD2	GNG8	PRKCA	DVL3	PFN1	NKD1
FZD3	GNGT1	PRKG1	FZD5	RAC1	NPHP3
FZD4	GNGT2	PRKG2	GRK5	RAC2	PLEKHA4
FZD5	ITPR1	TCF7	GSK3A	RAC3	RSPO3
FZD6	ITPR2	TCF7L1	GSK3B	RHOA	SFRP1
GNAO1	ITPR3	TCF7L2	KLHL12	ROR2	SFRP2
GNAT2	LEF1	TNRC6A	LRP6	RYK	ZNRF3
GNB1	MAP3K7	TNRC6B	NKD2	VANGL2	
GNB2	MOV10	TNRC6C	PI4K2A	WNT1	
GNB3	MYC	WNT11	PIP5K1B	WNT11	
GNB4	NFATC1	WNT5A	PPP2R5A	WNT4	
GNB5	NLK		RANBP3	WNT5A	
GNG10	PDE6A		WNT3A	WNT5B	
